# Patients with paradoxical low-flow, low-gradient aortic stenosis gain the least benefit from TAVI among all hemodynamic subtypes

**DOI:** 10.1007/s00392-024-02482-7

**Published:** 2024-07-02

**Authors:** Miriam Puls, Bo Eric Beuthner, Rodi Topci, Christoph Friedemann Jacob, Kristin Elisabeth Steinhaus, Niels Paul, Tim Beißbarth, Karl Toischer, Claudius Jacobshagen, Gerd Hasenfuß

**Affiliations:** 1 Clinic of Cardiology and Pneumology, University Medical Center Göttingen, 37099 Göttingen, Germany; 2 Department of Medical Bioinformatics, University Medical Center Göttingen, 37099 Göttingen, Germany; 3 Department of Cardiology, Vincentius-Diakonissen Hospital Karlsruhe, 76135 Karlsruhe, Germany

**Keywords:** Aortic stenosis; paradoxical low-flow, Low-gradient gradient aortic stenosis; TAVI

## Abstract

**Background:**

Substantial controversy exists regarding the clinical benefit of patients with severe paradoxical low-flow, low-gradient aortic stenosis (PLF-LG AS) from TAVI. Therefore, we compared post-TAVI benefit by long-term mortality (all-cause, CV and SCD), clinical improvement of heart failure symptoms, and cardiac reverse remodelling in guideline-defined AS subtypes.

**Methods:**

We prospectively included 250 consecutive TAVI patients. TTE, 6mwt, MLHFQ, NYHA status and NT-proBNP were recorded at baseline and 6 months. Long-term mortality and causes of death were assessed.

**Results:**

107 individuals suffered from normal EF, high gradient AS (NEF-HG AS), 36 from low EF, high gradient AS (LEF-HG), 52 from “classic” low-flow, low-gradient AS (LEF-LG AS), and 38 from paradoxical low-flow, low-gradient AS (PLF-LG AS).

TAVI lead to a significant decrease in MLHFQ score and NT-proBNP levels in all subtypes except for PLF-LG. Regarding reverse remodelling, a significant increase in EF and decrease in LVEDV was present only in subtypes with reduced baseline EF, whereas a significant decrease in LVMI and LAVI could be observed in all subtypes except for PLF-LG. During a follow-up of 3–5 years, PLF-LG patients exhibited the poorest survival among all subtypes (HR 4.2, *P* = 0.0002 for CV mortality; HR 7.3, *P* = 0.004 for SCD, in comparison with NEF-HG). Importantly, PLF-LG was independently predictive for CV mortality (HR 2.9 [1.3–6.9], *P* = 0.009).

**Conclusions:**

PLF-LG patients exhibit the highest mortality (particularly CV and SCD), the poorest symptomatic benefit and the least reverse cardiac remodelling after TAVI among all subtypes. Thus, this cohort seems to gain the least benefit.

**Graphical abstract:**

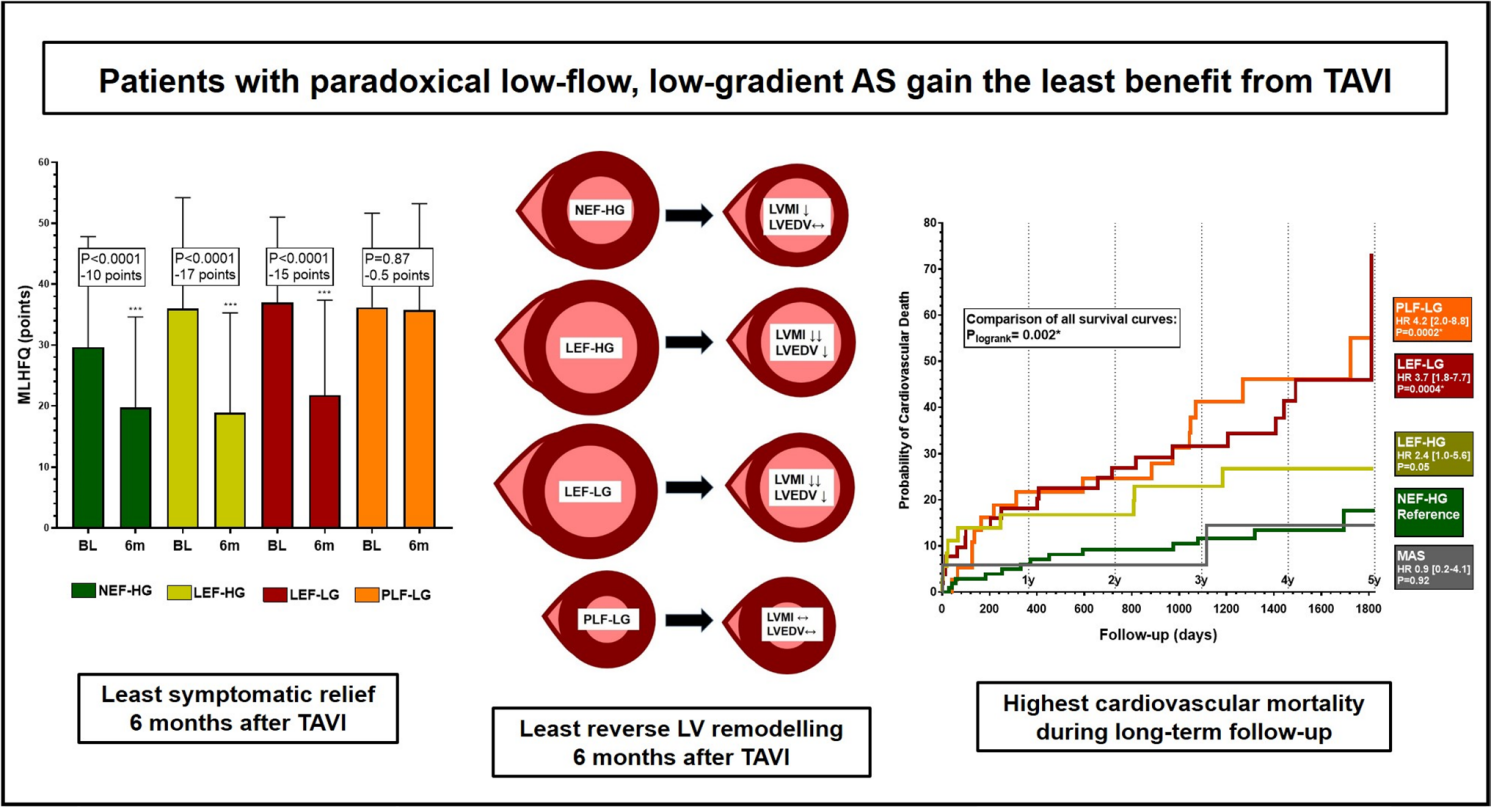

**Supplementary information:**

The online version contains supplementary material available at 10.1007/s00392-024-02482-7.

## Introduction

The entity of paradoxical low-flow, low-gradient aortic stenosis (PLF-LG AS) has long been under debate. The discordancy of calculated valve area and transvalvular gradients has frequently raised uncertainty with regard to the actual stenosis severity as well as the indications for intervention, particularly in the setting of preserved ejection fraction (EF). Hachicha et al. [1] were the first to describe that an important proportion of elderly patients with severe AS on the basis of AVA calculations tend to have low transvalvular gradients despite preserved EF. They found a significantly lower 3-year survival in the low-flow subgroup, defined by a stroke volume index (SVI) ≤ 35 ml/m^2^ BSA. Since then, further studies revealed many pathophysiological and clinical similarities between PLF-LG AS and HFpEF, i.e. the increasing prevalence with older age, female gender, and concomitant arterial hypertension [2].

A general treatment indication for severe symptomatic PLF-LG AS is commonly guideline-implemented all over the world, but the class of recommendation differs and the level of evidence remains low due to the lack of randomized trials in this subtype. Whereas American guidelines [3] provide a class 1-recommendation for all AS subtypes (class I A for severe high-gradient AS and class 1 B-NR for both classical and paradoxical low-gradient AS), European guidelines [4] differentiate differently: a class I B recommendation is given for high-gradient and classical low-gradient AS, and a lower class IIa C recommendation for PLF-LG AS. Thus, there is still substantial controversy and limited evidence regarding the clinical benefit of PLF-LG AS patients from TAVI. Therefore, we examined clinical improvement of heart failure symptoms, cardiac reverse remodelling, long-term mortality and causes of death in guideline-defined AS subtypes during post-TAVI follow-up.

## Methods

Between 1/2017 and 7/2019, we prospectively included 250 consecutive patients scheduled for transfemoral TAVI. Implantation was performed using standard techniques in a hybrid operating room (predominantly under general anaesthesia at that time). After careful evaluation in the interdisciplinary heart team, implantation strategy was changed to a transapical access in 4 patients. In the majority of cases (78%), the Sapien 3 valve (Edwards Lifesciences Inc., Irvine, CA, USA) was implanted. 43 patients (17%) received an Evolut Pro and 10 (4%) an Evolut R prosthesis (Medtronic, Minneapolis, MN, USA), and a Lotus valve (Boston Scientific, Marlborough, MA, USA) was implanted in a single case.

At baseline, transthoracic echocardiography (TTE), 6-min-walking test (6mwt), Minnesota Living with Heart failure Quality of life questionnaire (MLHFQ), NYHA status and NT-proBNP-levels were recorded. A structured follow-up visit repeating the baseline examinations was scheduled for 6 months. In addition, all patients were followed by regular telephone contact to assess mortality and causes of death (last in 6/2022).

The clinical benefit of TAVI was estimated by 3 components: (1) Clinical improvement of heart failure symptoms at six months (evaluated by NYHA status, MLHFQ points, NT-proBNP levels, and 6mwt distance); (2) Hemodynamic effects and reverse cardiac remodelling 6 months after TAVI (EF recovery; decrease of transvalvular gradients, LVEDV, LVMI, LAVI, and sPAP; evaluated by echocardiography); and (3) Long-term mortality and cause of death (all-cause, CV and SCD) during follow-up of 3–5 years for each individual patient. Regarding analysis of clinical changes after 6 months, only individuals with available paired observations were included. For definition of CV mortality and SCD, VARC-3 [5] and guideline definitions [6] were applied. The study was approved by the local ethics committee, and written informed consent was obtained from all patients.

### Echocardiography

All echocardiograms were performed using a Philips ie33 or a Philips Epiq7 system, and retrospectively reevaluated by a single observer using Q Station 3.8.5 (Philips healthcare). LV mass was calculated by the ASE-recommended cube formula (LV mass = 0.8 · 1.04 · [(IVSd + LVIDd + PWTd)^3^ − LVIDd^3^] + 0.6 g) using 2D linear LV measurements [7]. Subtype classification was performed according to a combination of P_mean_, LV-EF and SVI. Following current guidelines [3, 4], four subgroups of severe AS were defined:

1. Normal/ preserved ejection fraction, high-gradient AS (**NEF-HG AS**):
  $$EF\ge 50\%$$
  $${v}_{max}\ge 4 m/s\;or\; {P}_{mean}\ge 40 mmHg$$
  $$AVA \le 1.0 {cm}^{2}$$
2. Low/ reduced ejection fraction, high-gradient AS (**LEF-HG AS**):
  $$EF<50\%$$
  $${v}_{max}\ge 4 m/s\;or\;{P}_{mean}\ge 40 mmHg$$
  $$AVA \le 1.0 {cm}^{2}$$
3. Low/ reduced ejection fraction, low-gradient AS (“classic” low-flow, low-gradient AS) (**LEF-LG AS**):
  $$EF<50\%$$
  $${v}_{max}<4 m/s\;and\;{P}_{mean}<40 mmHg$$
  $$AVA\le 1.0 {cm}^{2}$$
  $$stroke\;volume\;index\;(SVI) \le 35 ml/{m}^{2}$$
4. Paradoxical low-flow, low-gradient AS (**PLF-LG AS**):
  $$EF \ge 50\%$$
  $${v}_{max}<4 m/s\;and\;{P}_{mean}<40\;mmHg$$
  $$AVA \le 1.0 {cm}^{2} and\;indexed\;AVA\;\le 0.6 {cm}^{2}/ {m}^{2}$$
  $$SVI \le 35 ml/ {m}^{2}$$

### Statistical analysis

Statistical analysis was performed with graph pad prism version 9.0 and with the Statistical Computing Software R (version 2.15.1; http://www.r-project.org). Continuous variables are presented as mean ± standard deviation and were compared using student’s t-test or Mann–Whitney U test for 2-group comparison (as appropriate). Categorical variables are presented as absolute numbers and percentage and were compared by Fisher’s exact test for 2-group comparison and by Pearson’s Chi-Square test for multi-group comparison. A value of *P* < 0.05 was considered statistically significant.

Survival analysis was performed on time from date of TAVI to event data for all-cause and CV mortality and SCD using the R package *survival*, visualized by Kaplan–Meier plots, and significance was calculated by the log rank test. A Benjamini-Hochberg-correction for multiple testing was performed. For multivariate models, the Cox Proportional Hazards Model was used. Survival analyses were performed for known outcome predictors according to literature or own previous publications. Only risk stratifiers that were found to be significant in univariate analyses were included in multivariate analyses.

## Results

Among the **250** study participants, **107** individuals suffered from **NEF-HG AS**, **36** from **LEF-HG AS**, **52** from **LEF-LG AS**, and **38** from **PLF-LG AS**. **17** patients who did not fulfil all criteria for one of these subgroups were retrospectively classified as “moderate-to-severe AS” (**MAS**). In these 17 MAS patients, decision for TAVI was nevertheless made after careful heart team evaluation due symptoms attributed to AS.

### Baseline demographic characteristics in AS subtypes

Baseline characteristics are displayed in Table 1. The total cohort (106 women, 144 men) was characterized by advanced age (mean, 79 ± 6 years) and a high comorbidity burden. 68% of patients pertained to NYHA classes III and IV.

**Table 1:** Baseline clinical characteristics

	MAS (*n* = 17)	NEF-HG AS (*n* = 107)	LEF-HG AS (*n* = 36)	LEF-LG AS (*n* = 52)	PLF-LG AS (*n* = 38)	*P* (comparison of all groups)
**Age,** *y*	78 ± 6	78 ± 6	79 ± 8	79 ± 6	81 ± 7	0.4
**Sex, female,** *n (%)*	5 (29)	57 (53)	12 (33)	10 (19)	22 (58)	**0.001**
**Diabetes,** *n (%)*	7 (41)	39 (36)	12 (33)	25 (48)	12 (32)	0.5
**Hypertension,** *n (%)*	16 (94)	99 (93)	29 (81)	48 (92)	36 (95)	0.2
**Coronary artery disease,** *n (%)*	14 (82)	64 (60)	24 (67)	42 (81)	26 (68)	0.07
**Prior MI,** *n (%)*	4 (24)	4 (4)	8 (22)	18 (35)	4 (11)	** < 0.0001**
**Prior PCI,** *n (%)*	5 (29)	24 (22)	6 (17)	24 (46)	16 (42)	**0.005**
**Prior CABG,** *n (%)*	6 (35)	8 (7)	1 (3)	13 (25)	1 (3)	** < 0.0001**
**Atrial fibrillation,** *n (%)*	8 (47)	28 (26)	13 (36)	25 (48)	28 (74)	** < 0.0001**
**Peripheral vascular disease**	4 (24)	10 (9)	3 (8)	15 (29)	4 (11)	**0.008**
**Prior cereb. ischem. event,** *n(%)*	2 (12)	17 (16)	3 (8)	11 (21)	7 (18)	0.6
**Chron. pulmon. disease,** *n (%)*	6 (35)	29 (27)	7 (19)	19 (37)	14 (37)	0.3
**Active malignant disease**	0	13 (12)	4 (11)	4 (8)	8 (21)	0.2
**CKD, GFR < 60 mL/min,** *n (%)*	9 (53)	50 (47)	16 (44)	32 (62)	22 (58)	0.4
**CKD, GFR < 30 mL/min,** *n (%)*	2 (12)	6 (6)	3 (8)	9 (17)	5 (13)	0.2
**Creatinine,** *mg/dl*	1.24 ± 0.5	1.11 ± 0.6	1.25 ± 0.8	1.65 ± 1.5	1.33 ± 0.8	**0.02**
**NT-proBNP,** *pg/ml*	1625 ± 1711	1650 ± 2285	8492 ± 8882	9444 ± 15,122	2279 ± 1623	** < 0.0001**
**MLHFQ,** *points*	29 ± 19	31 ± 18	36 ± 18	40 ± 15	38 ± 17	**0.02**
**6mwt distance,** *m*	305 ± 126	269 ± 103	200 ± 130	182 ± 123	206 ± 121	** < 0.0001**

Comparison of all 4 groups: 1way ANOVA for continuous variables; chi-square test for categorical variables

MI: myocardial infarction; PCI: percutaneous coronary intervention; CABG: coronary artery bypass grafting; CKD: chronic kidney disease; MLHFQ: Minnesota Living with Heart failure Quality of Life Questionnaire; 6mwt: six-minute walking test

Regarding the four AS subtypes, clear differences could be detected. Briefly, the low-gradient patients with reduced EF (**LEF-LG**) represented the sickest cohort with the highest prevalence of prior MI (35%), prior PCI (46%), and prior CABG (25%). Also, LEF-LG patients exhibited the most advanced signs of heart failure (highest MLHFQ total scores, highest NT-proBNP levels and lowest values for 6mwt distance among all subgroups). In the low-gradient cohort with preserved EF (**PLF-LG**), the proportion of women (58%) and the prevalence of atrial fibrillation (74%) were highest among all subgroups. Patients with high gradients and preserved EF (**NEF-HG**) were characterized by the lowest comorbidity burden.

### Echocardiographic LV remodelling and LV function in AS subtypes at baseline

Distinct patterns of baseline LV remodelling could be differentiated in the different AS subtypes (detailed depiction in suppl. table 1).

**NEF-HG** patients were characterized by a normal EF (60 ± 5%), an average normal LV cavity size (LVEDV_i_ 38 ± 12 ml/m^2^), and significant concentric LV hypertrophy (LVMI 138 ± 35 g/m^2^, RWT 0.68 ± 0.15). In contrast, **LEF-HG** patients (EF 36 ± 11%) showed significant LV dilatation (LVEDV_i_ 61 ± 17 ml/m^2^) and more advanced hypertrophy (LVMI with 173 ± 39 g/m^2^ highest among all subgroups; eccentric hypertrophy in 30% vs. 2% in NEF-HG, *P* < 0.0001), despite of similar transaortic gradients (P_mean_ 48 ± 12 vs. 47 ± 10 mmHg, *P* = 0.54). Thus, LEF-HG AS seems to represent a more advanced form of NEF-HG AS.

LV geometry of **LEF-LG** did not differ significantly from LEF-HG patients despite significantly lower transaortic gradients (P_mean_ 25 ± 7 vs. 47 ± 10 mmHg, *P* < 0.0001). Significant LV dilatation (LVEDV_i_ 65 ± 24 ml/m^2^ BSA) and LV hypertrophy (LVMI 169 ± 42 g/m^2^ BSA) were present, with the lowest RWT (0.48 ± 0.11) and the highest rate of eccentric hypertrophy (40%) of all subgroups. In contrast to LEF-HG, patients’ history documented the development of severe AS in a pre-existing failing LV in many cases.

**PLF-LG** patients had the smallest LV cavity size (LVEDV_i_ 35 ± 11 ml/m^2^) and the lowest LVMI among all subgroups (131 ± 38 g/m^2^), yet indicating concentric remodelling in 13% and concentric hypertrophy in 87% of patients (RWT 0.62 ± 0.10). Although LV-EF was preserved (58 ± 6%), global longitudinal strain suggested subtle dysfunction (GLS -17.6 ± 3%). PLF-LG patients exhibited high left atrial volumes (LAVI 54 ± 16 ml/m^2^, higher than LVEDV_i_) and the highest prevalence of atrial fibrillation during echo recording (55%). Thus, impaired LV filling seems to be the major determinant of reduced SVI in this subtype.

### TAVI procedure and procedural complications

244 patients were treated via transfemoral and 4 via transapical access (1 NEF-HG, 3 LEF-LG patients). Two individuals died shortly before scheduled (transfemoral) procedure. Detailed procedural data and complications in the different subtypes are summarized in suppl. Table 2. Neither distribution of implanted valves nor total procedural duration, fluoroscopy time, dose-area-product or amount of contrast agent differed significantly between AS subtypes. Regarding procedural complications, the incidence of bleeding complications, access complications, acute kidney injury, new-onset conduction disturbances including new pacemaker implantation, pericardial tamponade, coronary obstruction, and myocardial infarction did not differ significantly either. Exclusively, the 5 cases of stroke/ TIA were not equally distributed (2 LEF-HG and 3 PLF-LG patients, *P* = 0.01). Six patients died before first discharge after TAVI (1 NEF-HG, 2 LEF-HG, 2 LEF-LG, 0 PLG-LG, and 1 MAS).

## 6-months follow-up clinical visit

The 6 months follow-up (median, 195 days) was 73% complete (183/250 patients): 29 patients (12%) had already died, 10 persons (4%) were too sick to attend and 28 individuals (11%) refused the clinical visit. The NYHA class could be collected by telephone call in nine further patients (total *n* = 192).

### Clinical improvement of heart failure symptoms 6 months after TAVI

**Fig. 1:**
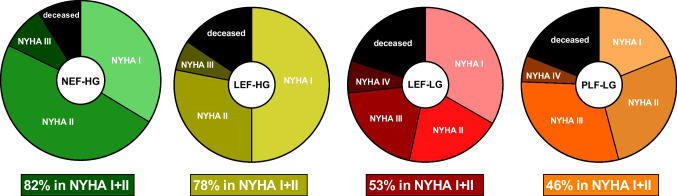
NYHA status (including the status “deceased”) in different AS subtypes at 6 months after TAVI. 82% of NEF-HG, but only 46% of PLF-LG patients pertained to NYHA classes I and II

NYHA-status at follow-up differed significantly between AS subtypes (*P* < 0.0001, Fig. 1). To minimize positive selection bias due to death of the sickest patients, we added the class “deceased”. Six months after TAVI, as many as 54% of PLF-LG patients (but only 18% of NEF-HG patients) were dead or in NYHA classes 3 or 4.

**Fig. 2:**
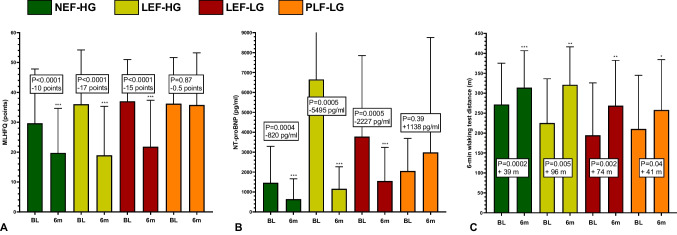
Development of symptomatic heart failure burden from baseline to 6 months after TAVI in different AS subtypes; development of **A** MLHFQ score, **B** NT-proBNP-levels, and **C** 6-min walking test distances. A significant improvement of MLHFQ score and NT-proBNP-levels was present in all subtypes except for PLF-LG AS

In the surviving patients who attended 6 months follow-up, we observed a significant improvement of subjective heart failure burden (by MLHFQ score) for NEF-HG (-10 points, *P* < 0.0001), LEF-HG (-17 points, *P* < 0.0001) and LEF-LG patients (-15 points, *P* < 0.0001), whereas MLHFQ score did not change in PLF-LG patients (*P* = 0.87). Similarly, NT-proBNP levels decreased significantly in all hemodynamic subtypes except for PLF-LG AS. However, an increase in 6mwt distances was present in all subgroups (NEF-HG: + 39 m, *P* = 0.0002; LEF-HG: + 96 m, *P* = 0.0005; LEF-LG: + 74 m, *P* = 0.0002; PLF-LG: + 41 m, *P* = 0.04) (Fig. 2A-C).

### Hemodynamic effects and reverse cardiac remodelling 6 months after TAVI

**Fig. 3:**
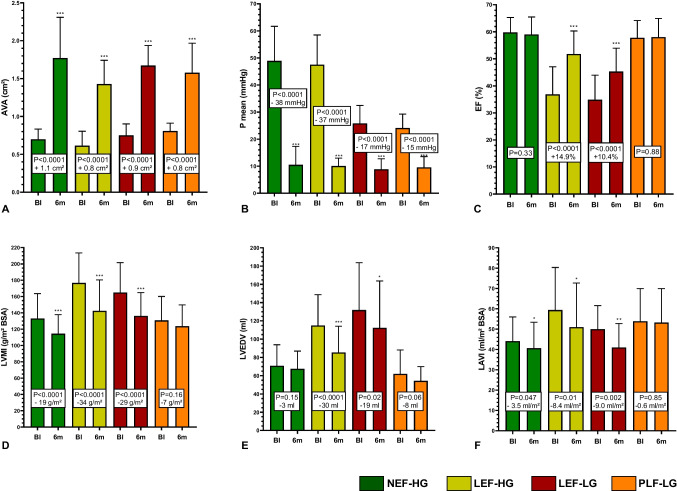
Hemodynamic effects and reverse left ventricular remodelling 6 months after TAVI compared to baseline in different AS subtypes; development of **A** aortic valve area (AVA), **B** mean transvalvular gradient (P_mean_), **C** LV ejection fraction (EF), **D** left ventricular mass index (LVMI),** E** left ventricular end-diastolic volumes (LVEDV), and **F** left atrial volume index (LAVI). A significant decrease of LVMI and LAVI could be observed in all AS subtypes except for PLF-LG. Subtypes with reduced baseline EF additionally demonstrated a significant EF increase and LVEDV decrease

For detailed depiction of all changes in echo parameters from baseline to 6 months in the different subgroups, see also suppl. table 3. TAVI significantly increased AVA in all subgroups (Fig. 3A). Mirroring the different baseline remodelling patterns, we also observed significant differences in reverse LV remodelling between AS subtypes (Fig. 3B-F).

In **NEF-HG AS**, the greatest reduction of transvalvular gradients among all subgroups (-38 ± 15 mmHg, *P* < 0.0001) was achieved by TAVI. This afterload reduction lead to a significant regression of RWT (*P* = 0.008) and LVMI (-19 ± 24 g/m^2^, *P* < 0.0001). Also, a decrease of LAVI (-3.5 ± 14 ml/m^2^, *P* = 0.047) and of moderate or severe MR prevalence (14% at 6 months vs. 35% at BL, *P* = 0.002) could be noticed.

In **LEF-HG AS**, the immense afterload reduction by AS elimination (P_mean_—37 ± 11 mmHg compared to BL, *P* < 0.0001) lead to significant LV unloading. We saw the greatest regression of LV volumes (LVEDV: -30 ± 26 ml; LVESV: -32 ± 24 ml), LV diameters (LVEDD: -5.3 ± 5.6 mm; LVESD: -6.4 ± 7.7 mm; *P* < 0.0001), and LV hypertrophy (LVMI: -34 ± 20 g/m^2^, *P* < 0.0001) among all subtypes. In addition, LV-EF nearly normalized (52 ± 13% vs. 37 ± 10% at BL, *P* < 0.0001), LAVI decreased by 8.3 ± 13.5 ml/m^2^ (*P* = 0.01) and sPAP by 10 ± 11 mmHg (*P* = 0.002).

Reverse remodelling in **LEF-LG AS** resembled the pattern in LEF-HG, although the reduction of transvalvular gradients after TAVI was less pronounced (-17 ± 8 mmHg, *P* < 0.0001): LVEDV decreased by 19 ± 42 ml (*P* = 0.02), LVEDD by 4.0 ± 5.0 mm (*P* < 0.0001), and LVMI by 29 ± 29 g/m^2^ (*P* < 0.0001). LV-EF also recovered significantly by 10 ± 11% (45 ± 12% vs. 35 ± 9% at BL, *P* < 0.0001), but less pronounced than in LEF-HG. However, TAVI-induced LV unloading lead to a significant decrease of LAVI (- 8.8 ± 12 ml/m^2^, *P* = 0.002) and sPAP (- 12 ± 16 mmHg, *P* = 0.003).

The least reduction of transvalvular gradients after TAVI was observed in **PLF-LG AS** (-14.5 ± 8 mmHg), *P* < 0.001). At 6 months, we observed no significant changes regarding LV volumes (trend towards reduction: LVEDV 62 ± 26 ml vs. 54 ± 1 ml, P = 0.06), LV diameters (LVEDD 43.1 ± 5 mm vs. 42.2 ± 5 mm, *P* = 0.37), LVMI (131 ± 29 vs. 124 ± 26 g/m^2^, *P* = 0.16), and LAVI (54 ± 16 vs 53 ± 17 ml/m^2^, *P* = 0.85). Of note, diastolic function as measured by E/e’ did not improve at all (15.10 ± 5.5 vs. 15.96 ± 6.6, *P* = 0.98).

However, a trend towards GLS increase was present (-16.8 ± 2.5% vs. -17.8 ± 2%, *P* = 0.053). The only parameter with significant improvement in PLF-LG patients was sPAP (- 12 ± 22 mmHg, *P* = 0.03).

To elucidate determinants and effects of reverse LV remodelling, we correlated absolute LVMI change (g/m^2^) with other variables. A greater LVMI decrease highly significantly (*P* < 0.001) correlated with higher baseline LVMI (spearman r =—0.61), higher baseline LVEDV (r =—0.40), higher baseline NT-proBNP levels (r = -0.34), and lower baseline EF (r = 0.26). Taken together, the dilative remodelling type exhibited a greater potential for LVMI regression. However, also higher baseline transvalvular gradients (r =—0.21, *P* = 0.005) and greater P_mean_ reduction at 6 months (r = 0.20, *P* = 0.006) were associated with greater LVMI regression. Furthermore, LVMI regression significantly correlated with LAVI decrease (r = 0.27, *P* = 0.001), but not with sPAP decrease (r = 0.15, *P* = 0.11).

### Correlation of clinical and echocardiographic recovery at 6 months

A more pronounced regression of symptomatic heart failure burden (evaluated by absolute decrease in MLHFQ score) correlated significantly with the absolute decrease in NT-proBNP-levels (r = 0.28, *P* = 0.002), the absolute increase of LV-EF (r = -0.24, *P* = 0.001), absolute increase in 6mwt distance (r = -0.21, P = 0.02) and absolute decrease in LVMI (r = 0.17, *P* = 0.03). In contrast, the absolute decrease of P_mean_ did not correlate with symptomatic relief (*P* = 0.62).

## Long-term mortality and causes of death

Two hospitalised patients died from AS before the scheduled TAVI. VARC-3[5]-defined periprocedural mortality (death that occurs within 30 days of the index procedure or beyond 30 days if the patient is still hospitalised) was 3.6% (6% in MAS, 1% in NEF-HG, 8% in LEF-HG, 6% in LEF-LG, and 3% in PLF-LG AS; *P* = 0.22).

Follow-up period after TAVI ranged between 3 and 5 years. In total, 107/250 patients (43%) had died at time of last follow-up. Non-cardiovascular deaths (*n* = 47) most frequently occurred due to malignant diseases (40%), pneumonia including COVID (23%), or other septicaemia (11%). However, 60/107 deaths were classified as cardiovascular (CV): Although TAVI was performed in order to prevent CV death, as many as 20/250 (8%) of our patients died from terminal heart failure, and 24/250 (10%) from SCD during long-term follow-up.

**Fig. 4:**
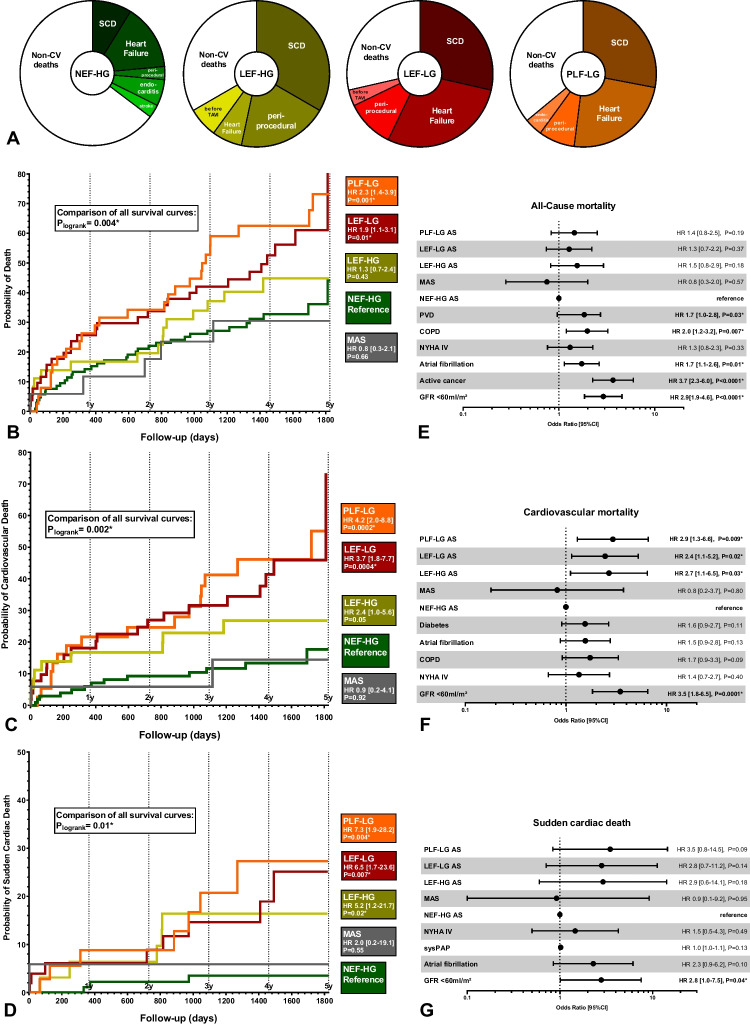
Survival during long-term follow-up after TAVI in different AS subtypes. **A** Ratio of different causes of death in different AS subtypes**. B-D:** Kaplan–Meier-curves displaying all-cause mortality (B), cardiovascular mortality (C), and sudden cardiac death (D) in different AS subtypes. **E–G:** Odds ratio plots displaying multivariate cox regression analyses for prediction of all-cause mortality (E), cardiovascular mortality (F) and sudden cardiac death (G). Unexpectedly, SCD was the most frequent cause of CV death in all subtypes except for NEF-HG AS. In comparison with NEF-HG, PLF-LG patients exhibited the most pronounced relative risk elevations for total mortality, cardiovascular mortality and sudden cardiac death during long-term follow-up. PLF-LG, LEF-LG, and LEF-HG status were independently predictive for CV death. **NEF-HG**: normal EF, high gradient; **LEF-HG**: reduced EF, high gradient; **LEF-LG**: reduced EF, low gradient (classic low-flow, low-gradient); **PLF-LG**: paradoxical low-flow, low-gradient; **MAS**: moderate-to-severe AS

Total follow-up mortality varied significantly between AS subtypes (29% in MAS, 32% in NEF-HG, 42% in LEF-HG, 54% in LEF-LG, and 66% in PLF-LG AS patients, *P* = 0.002). CV mortality rates were also significantly different (*P* < 0.0001): 2/17 (12%) in MAS, 12/107 (11%) in NEF-HG AS, 10/36 (28%) in LEF-HG AS, 20/52 (38%) in LEF-LG AS, and 16/38 (42%) in PLF-LG AS. Of note, not only survival rates, but also causes of deaths differed between subtypes (Fig. 4A): Whereas death was of non-CV origin in 2/3 of NEF-HG patients, it was cardiovascular in 2/3 of all other patients. Importantly, sudden cardiac death (SCD) was the most common CV death among all subtypes except for NEF-HG.

### Survival analyses

Kaplan–Meier-curves for all-cause mortality, CV mortality and SCD are displayed in Fig. 4B-D. Compared with NEF-HG as reference, all-cause mortality was significantly elevated in PLF-LG (HR 2.33 [1.4–3.9], *P* = 0.001) and LEF-LG (HR 1.88 [1.1–3.1], *P* = 0.01) (Fig. 4B). In a multivariate Cox-regression model however, independent risk factors for all-cause mortality were only active cancer, renal insufficiency with GFR < 60 ml/min/1.73 m^2^, COPD, atrial fibrillation, and peripheral vascular disease (Fig. 4E).

Regarding cardiovascular (CV) mortality, survival differences between AS subtypes were even more pronounced, with the significantly lowest rate for NEF-HG in comparison with all other high-grade subtypes (Fig. 4C). Multivariate analysis confirmed the AS subtypes LEF-HG, LEF-LG, and PLF-LG AS, as well as GFR < 60 ml/min/1.73 m^2^ as independent predictors of CV mortality after TAVI (Fig. 4F).

We furthermore analysed SCD as most frequent cause of death (Fig. 4D). Survival analyses again revealed the lowest rate for NEF-HG in comparison with all other high-grade subtypes. However, in multivariate analysis only GFR < 60 ml/min/1.73 m^2^ remained predictive, probably due to the relatively low event rate for this single diagnosis (Fig. 4G). However, PLF-LG AS nearly gained significance (*P* = 0.09).

## Discussion

The aim of our study was to evaluate the clinical benefit of TAVI (defined by improvement of heart failure signs and symptoms, reverse LV remodelling and survival) in different hemodynamic AS subtypes.

The main findings are: 

1. A significant improvement of subjective heart failure burden as estimated by significant decrease of MLHFQ scores and NT-proBNP-levels was observed in all AS subtypes except for PLF-LG AS. Treatment futility as indicated by death or NYHA functional class III or IV was present in as much as 54% of PLF-LG patients at 6 months, but only in 18% of NEF-HG patients.
2. Regarding reverse LV remodelling, a significant decrease of LVMI and LAVI was present in all hemodynamic AS subtypes except for PLF-LG 6 months after TAVI. Subtypes with reduced baseline EF additionally demonstrated a significant EF increase and LVEDV decrease.
3. In comparison with NEF-HG, PLF-LG patients exhibited significantly higher risks of total mortality (HR 2.3, *P* = 0.001), cardiovascular mortality (HR 4.2, *P* = 0.0004), and sudden cardiac death (HR 7.3, *P* = 0.004) during long-term follow-up of up to 5 years. The relative risk elevations were less pronounced in all other subtypes. Unexpectedly, SCD was the most frequent cause of CV death in PLF-LG. PLF-LG, LEF-LG, and LEF-HG status were independently predictive for CV death, with the highest hazard ratio for PLF-LG AS (2.9 [1.3–6.9], *P* = 0.009).
4. In conclusion, among all AS subtypes, PLF-LG patients exhibited the least symptomatic improvement, the least reverse remodelling, and the highest cardiovascular mortality during follow-up after TAVI. Thus, PLF-LG patients gained the least benefit from TAVI.

### Clinical benefit

Few studies exclusively focus on the clinical benefit of PLF-LG patients after TAVI. Rodriguez-Gabella et al.[8] observed TAVI treatment futility (defined as death or NYHA functional class III or IV at 6-month follow-up) significantly more frequently in 97 PLF-LG compared to 396 NEF-HG patients (24% vs 14%; *P* < 0.001). In line with these results, the same endpoint occurred in even 54% of PLF-LG vs. 17.8% of NEF-HG patients (*P* < 0.0001) in our patient cohort. Predictors of treatment futility (similarly defined as all-cause mortality, NYHA class III-IV or deterioration in functional class at 1-year follow-up) were investigated by Freitas-Ferraz et al.[9] in 318 PLF-LG patients. This endpoint was observed in one third of their patients and could be predicted by the presence of atrial fibrillation, COPD and SVI < 30 ml/m^2^. Analyses in our own PLF-LG cohort also revealed a higher prevalence of SVI < 30 ml/m^2^ (60% vs. 17.6%, *P* = 0.02), a higher baseline MLHFQ score (43 ± 16 vs. 31 ± 14, *P* = 0.03), and a lower baseline 6mwt distance (156 ± 104 vs. 265 ± 109 m, *P* = 0.008) in PLF-LG patients with treatment futility. None of the 5 patients in NYHA class 4 at baseline showed clinical improvement, and 4 of them died within the first year after TAVI. Prevalence of atrial fibrillation (70% vs. 76%, *P* = 0.72) or COPD (30% vs. 12%, P = 0.25) did not differ significantly.

Several studies report NYHA status only in surviving patients after a longer follow-up period after TAVI. Two large single-centre registries including 1600 [10] and 1800 patients [11] reported significantly higher mortality rates at 3 years after TAVI in PLF-LG patients compared with HG patients, but surviving patients showed a similar improvement in NYHA class regardless of AS entity. However, the status “deceased” was not included in the analysis, and therefore a positive selection bias due to death of the sickest patients has to be postulated.

In our own PLF-LG patients, we nevertheless found an increase in 6mwt distances at 6 months (+ 41 m, *P* = 0.04). 66% of patients with increased walking distances vs. only 17% without 6mwt improvement were male (*P* = 0.04). In summary, a clinical benefit of TAVI in PLF-LG AS seems more likely in patients in less advanced stages of heart failure, and in men.

### Reverse LV remodelling

Reports concerning reverse LV remodelling in PLF-LG patients after TAVI are still scarce and controversial. Dahou et al. [12] compared pre-operative and 1-year echocardiography of 32 patients with PLF-LG AS (no comparisons with other subtypes). At 1 year, a significant increase in LVEDD and LVEDV with a decrease in septum and posterior wall thickness could be observed, resulting in a significant decrease in LV mass (207 vs. 175 g; *p* = 0.002). While LVEF remained unchanged, GLS increased significantly from baseline to 1 year (-14.5 ± 3.9% vs. -17.2 ± 4.0%; *p* = 0.03). In our own PLF-LG patient cohort, neither wall thickness, LVEDD, LVEDV nor LVMI changed significantly, and only a trend towards GLS increase could be observed (-16.8 ± 2.5% vs. -17.8 ± 2%, *P* = 0.053). Similar to our own results, Kamperidis et al. [13] found differences in functional recovery after TAVI between 35 LEF-LG and 33 PLF-LG patients 6 and 12 months after TAVI. Whereas all functional parameters improved in LEF-LG AS (like in our own cohort), only strain-derived parameters significantly improved in PLF-LG AS. LV volumes decreased significantly in LEF-LG but not in PLF-LG.

### Prognosis

The majority of previous studies reported worse spontaneous [14, 15] or post-interventional [10, 11, 15–17] outcomes of PLF-LG patients in comparison with HG AS. A meta-analysis including 7459 patients from 18 studies [14] performed an observational comparison of AVR versus conservative management in AS patients. Compared with NEF-HG, PLF-LG patients exhibited a 1.67 fold increase of total mortality under conservative management, and AVR reduced mortality by 57%. A second meta-analysis including 27,204 TAVI patients from 19 studies [16] focused on post-TAVI outcomes and reported higher mortality rates for LEF-LG and PLF-LG patients in comparison with HG patients, whereas outcomes in both LG subgroups did not differ. Similarly, in the OCEAN-TAVI registry PLF-LG was associated with a 3.76 fold increased all-cause mortality as compared with NEF-HG patients during a follow-up of 18 months after TAVI [17].

In contrast, a recent analysis of the PARTNER 2 trial and registry [18] did not find a higher follow-up mortality in PLF-LG compared with NEF-HG AS. However, both PARTNER trials were not designed to investigate low-gradient AS. Per inclusion criteria, they were designed to enrol only HG patients, but the corelab confirmed the presence of HG status in only two-thirds of patients.

### To match or not to match – Is that the question?

Given the consistently differing baseline characteristics of LG and HG patients, some authors raised the question if worse outcomes of LG patients might be a consequence of comorbidity burden rather than of AS subtype itself. Thus, Fischer-Rasokat et al. [19] compared outcomes between HG, LEF-LG and PLF-LG patients with and without propensity score matching, and found no significant survival differences between matched HG and PLF-LG patients (*p* = 0.47). Similarly, Mosleh et al. [20] investigated the hemodynamic and clinical benefit of TAVI in 73 PLF-LG patients compared with 217 HG patients after propensity score matching for age, comorbidities (including diabetes, hypertension, presence of atrial fibrillation, prior stroke), baseline NYHA class, baseline LVEF, and STS score. Again, no differences in mortality, improvement in NYHA class or Kansas City Cardiomyopathy Questionnaire score could be found in the matched cohorts.

In summary, most previous analyses in unmatched patient cohorts reported worse survival and less functional recovery after TAVI in PLF-LG if compared with NEF-HG patients, whereas analyses in propensity score matched cohorts reported similar outcomes. In generally, propensity score matching is a technique for constructing an artificial control group in non-randomized studies that nevertheless aim at evaluating the effect of a medical intervention. Since we do not compare a study cohort that received an intervention with another that did not, but the effects of an intervention in different disease subtypes, the question of appropriateness of this statistical method should be raised. As increasingly acknowledged, the LV response is heterogeneous and multiple phenotypes of LV remodelling in AS exist [21, 22]. It seems most plausible that a distinct clinical profile induces the distinct remodelling pattern of PLF-LG patients, which are frequently reported to be of older age, often female, and exhibit a higher prevalence of atrial fibrillation and smaller LV cavity sizes in comparison with NEF-HG AS. Particularly atrial fibrillation is probably not an innocent bystander, but a potential reason for low gradients in patients with normal EF. Thus, matching for comorbidities might also level out the underlying pathophysiology. In our analysis, we decided to perform a multivariate analysis allowing to estimate the effect of the hemodynamic AS subtype while normalizing for the effects of all other predictors. Accordingly, PLF-LG status emerged as independent predictor of cardiovascular mortality, whereas all other mentioned risk factors did not (Fig. 4F).

Therefore, risk evaluation according to AS subtypes seems a reasonable approach for daily clinical practice in our eyes. Otherwise, we might miss the chance to identify further treatment options to improve prognosis in this specific patient cohort which shares so many features with HFpEF. In fact (and as demonstrated by absent recovery of diastolic function), many PLF-LG AS patients stay symptomatic HFpEF patients with all typical features after TAVI. In a previous publication (including a part of the current study cohort), we investigated biopsy-derived myocardial fibrosis in different AS subtypes [23]. Baseline histological myocardial fibrosis was also quantified in 116 patients of the current study cohort. Median fibrosis burden was generally elevated and differed significantly between subtypes (*P* = 0.03: 9.2% in NEF-HG, 16.4% in LEF-HG, 25.8% in LEF-LG, 11.2% in PLF-LG, and 9.4% in MAS). Absence of reverse LV remodelling after TAVI in PLG-LG AS might suggest that AS is not the (only) stimulus leading to myocardial fibrosis in this entity. Thus, SGLT2-inhibition or anti-fibrotic principles might be promising approaches. The recognition of PLF-LG AS patients as HFpEF patients would probably lead to improved treatment in this cohort, since options like HFpEF medication or catheter ablation of atrial fibrillation would more likely be considered.

## Conclusions

Among all AS subtypes, PLF-LG patients gained the least benefit from TAVI in terms of clinical improvement, reverse LV remodelling and cardiovascular mortality. Many of them stay symptomatic HFpEF patients after TAVI. Thus, the recognition as HFpEF patients with all therapeutic consequences could represent a major step towards improved outcomes in this subtype.

### Limitations

Our analysis represents a single-centre experience with a relatively small sample size and can therefore only be considered as hypothesis generating. However, the strength lies in the high completeness of follow-up (100% for survival status). Study patients included in the manuscript were enrolled between 1/2017 and 7/2019, so clinical visits at 6 months were complete before the first COVID lockdown. Our registry did not stop during the pandemic, but the performance of clinical visits became very difficult. Therefore, we decided not to include the later cohort in the present analysis. We cannot exclude that COVID may have affected mortality in our patients, but we can assume that it did not affect distribution of deaths between AS subtypes according to our data.

## Supplementary information

Below is the link to the electronic supplementary material.

Supplementary file1 (DOCX 49 KB)

## Abbreviations

AS: Aortic stenosis
NEF-HG AS: Normal/ preserved ejection fraction, high-gradient AS
LEF-HG AS: Low/ reduced ejection fraction, high-gradient AS
LEF-LG AS: Low/ reduced ejection fraction, low-gradient (“classic” low-flow, low-gradient) AS
PLF-LG AS: Paradoxical low-flow, low-gradient AS
MAS: Moderate-to-severe AS
