# Supplementary material for: Patients with paradoxical low-flow, low-gradient aortic stenosis gain the least benefit from TAVI among all hemodynamic subtypes
Source: Clin Res Cardiol. 2024 Jul 2;114(11):1479–90. doi: 10.1007/s00392-024-02482-7 (PMC12540541; doi:10.1007/s00392-024-02482-7)
Supplement: Supplementary file 1 — Supplementary file1 (DOCX 49 KB) [file 392_2024_2482_MOESM1_ESM.docx]

**Suppl. Table 1: Baseline echocardiographic characteristics**

|  | | **NEF-HG AS**  **(n=107)** | **LEF-HG AS (n=36)** | **LEF-LG AS (n=52)** | **PLF-LG AS (n=38)** | **MS AS**  **(n=17)** | **P (comparison of all groups)** |
| --- | --- | --- | --- | --- | --- | --- | --- |
|  |  | |  |  |  |  |  |
| **Afib during echo,** *n (%)* | 13 (12%) | | 7 (19%) | 17 (33%) | 21 (55%) | 5 (29%) | **<0.0001** |
| **LV-EF,** *%* | 60±5 | | 36±11 | 33±10 | 58±6 | 54.4±11 | **<0.0001** |
| **Global long. strain,** *%* | -18.3±2.7 | | -10.8±3.6 | -9.7±3.2 | -17.6±2.9 | -16.7±3.3 | **<0.0001** |
| **LVEDV,** *ml* | 72±26 | | 120±38 | 128±48 | 65±56 | 89±53 | **<0.0001** |
| **LVEDV_i_,** *ml/m² BSA* | 38±12 | | 61±17 | 65±24 | 35±11 | 47±27 | **<0.0001** |
| **Stroke Volume Index,** *ml/m²* | 41.9±10 | | 34.7±9 | 29.4±6.9 | 30.3±4.1 | 42.3±7.4 | **<0.0001** |
| **LAVI,** *ml/m² BSA* | 47±15 | | 58±19 | 54±15 | 54±16 | 49±14 | **0.003** |
| **LVMI,** *g/m² BSA* | 138±35 | | 173±39 | 169±42 | 131±31 | 144±43 | **<0.0001** |
| **LVMI/ LVEDV_i_,** *g/ml* | 3.8±1.2 | | 3.0±0.8 | 2.8±0.8 | 4.0±1.2 | 3.6±1.3 | **<0.0001** |
| **LVEDD,** *mm* | 42.4±6.4 | | 51.9±8.6 | 54.3±7.6 | 43.2±5.3 | 45.8±8.7 | **<0.0001** |
| **Septal wall,** *mm* | 16.1±2.4 | | 15.8±2.0 | 14.9±2.5 | 14.7±2.1 | 15.2±1.9 | **0.003** |
| **Posterior wall,** *mm* | 14.1±2.2 | | 13.7±2.6 | 12.8±2.2 | 13.3±1.8 | 13.6±1.7 | **0.009** |
| **Relative wall thickness** | 0.68±0.15 | | 0.55±0.17 | 0.48±0.11 | 0.62±0.10 | 0.62±0.15 | **<0.0001** |
| **Concentric remodelling,** *n (%)* | 14 (13%) | | 1 (3%) | 0 | 5 (13%) | 3 (18%) | **0.03** |
| **Concentric hypertrophy,** *n (%)* | 91 (85%) | | 24 (67%) | 31 (60%) | 33 (87%) | 13 (76%) | **0.001** |
| **Eccentric hypertrophy,** *n (%)* | 2 (2%) | | 11 (30%) | 21 (40%) | 0 | 1 (6%) | **<0.0001** |
| **E/e’ mean** | 17.4±6.5 | | 19.4±7.2 | 14.9±4.3 | 15.0±1.0 | 17.0±4.3 | 0.14 |
| **V_max_,** *m/s* | 4.5±0.5 | | 4.4±0.4 | 3.3±0.4 | 3.2±0.3 | 3.4±0.5 | **<0.0001** |
| **Mean Gradient,** *mmHg* | 48±12 | | 47±10 | 25±7 | 24±6 | 25±8 | **<0.0001** |
| **Aortic valve area (AVA),** *cm²* | 0.70±0.17 | | 0.62±0.15 | 0.76±0.16 | 0.76±0.14 | 0.99±0.14 | **<0.0001** |
| **Indexed AVA,** *cm²/m² BSA* | 0.38±0.09 | | 0.32±0.08 | 0.37±0.41 | 0.40±0.46 | 0.52±0.08 | **<0.0001** |
| **PAPs,** *mmHg* | 42.3±14.2 | | 54±17 | 47±13 | 51±17 | 48±20 | **0.004** |
| **TAPSE,** *mm* | 22.4±4.3 | | 19.9±4.6 | 17.5±3.7 | 19.1±4.3 | 17.5±4.3 | **<0.0001** |
| **Moderate or severe MR,** *n (%)* | 39 (36%) | | 20 (56%) | 38 (73%) | 21 (55%) | 7 (41%) | **0.001** |
| **Moderate or severe TR,** *n (%)* | 21 (20%) | | 13 (36%) | 21 (40%) | 19 (50%) | 13 (76%) | **<0.0001** |
|  |  | |  |  |  |  |  |

- Comparison of all 4 groups: 1way ANOVA for continuous variables; chi-square test for categorical variables
- LV-EF: left ventricular ejection fraction; LVEDV: left ventricular end-diastolic volume; BSA: body surface area; SVI: stroke volume index; LAVI: left atrial volume index; LVMI: left ventricular mass index; LVEDD: left ventricular end-diastolic diameter; RWT: relative wall thickness; GLS: global longitudinal strain; v_max_: maximum aortic velocity; AVA: aortic valve are; AVAi: indexed aortic valve area, AVA/BSA; PAsP: pulmonary artery systolic pressure; TAPSE: tricuspid annular plane systolic elevation; MR: mitral regurgitation; TR: tricuspid regurgitation

**Suppl. table 2. Perioperative Outcome (VARC-3 definitions)**

| **Procedural parameters** | **Total cohort (n=250)** | **NEF-HG AS**  **(n=107)** | **LEF-HG AS (n=36)** | **LEF-LG AS (n=52)** | **PLF-LG AS (n=38)** | **MAS**  **(n=17)** | **P (comparison of all groups)** |
| --- | --- | --- | --- | --- | --- | --- | --- |
|  |  |  |  |  |  |  |  |
| **Transapical access *(n, %)*** | 4 (2) | 1 (1) | 0 | 3 (6) | 0 | 0 | 0.11 |
|  |  |  |  |  |  |  |  |
| **Sapien 3 *(n, %)*** | 194 (78) | 83 (78) | 30 (83) | 43 (83) | 27 (71) | 11 (65) | 0.40 |
| **Evolut Pro *(n, %)*** | 43 (17) | 21 (20) | 4 (11) | 4 (8) | 10 (26) | 4 (24) | 0.12 |
| **Evolut R *(n, %)*** | 10 (4) | 3 (3) | 1 (3) | 4 (8) | 1 (3) | 1 (6) | 0.60 |
| **Lotus *(n, %)*** | 1 (0.4) | 0 | 0 | 0 | 0 | 1 (6) | NA |
| **Died before procedure *(n, %)*** | 2 (1) | 0 | 1 (3) | 1 (2) | 0 | 0 | 0.42 |
|  |  |  |  |  |  |  |  |
| **Total procedural duration (min)** | 87±42 | 89±46 | 99±47 | 76±32 | 85±39 | 82±37 | 0.34 |
| **Fluoroscopy time (min)** | 10.7±5.1 | 10.8±5.6 | 10.5±1.6 | 10.9±5.1 | 10.7±4.3 | 9.5±4.8 | 0.69 |
| **Dose-area-product (Gycm²)** | 38±25 | 38±29 | 42±22 | 38±18 | 36±26 | 31±14 | 0.21 |
| **Amount contrast agent (mL)** | 141±38 | 143±40 | 149±46 | 131±25 | 143±40 | 133±26 | 0.55 |
|  |  |  |  |  |  |  |  |
| **Conversion to surgical AVR** | 0 | 0 | 0 | 0 | 0 | 0 | NA |
| **Unplanned use of cardiopulm. bypass** | 0 | 0 | 0 | 0 | 0 | 0 | NA |
| **Ventricular perforation** | 0 | 0 | 0 | 0 | 0 | 0 | NA |
| **Pericardial tamponade** | 2 (1) | 1 (1) | 1 (3) | 0 | 0 | 0 | 0.61 |
| **“Valve-in-valve”** | 0 | 0 | 0 | 0 | 0 | 0 | NA |
| **Coronary obstruction** | 2 (1) | 2 (2) | 0 | 0 | 0 | 0 | 0.60 |
|  |  |  |  |  |  |  |  |
| **Myocardial infarction*** | 2 (1) | 2 (2) | 0 | 0 | 0 | 0 | 0.60 |
| **Stroke*** | 4 (2) | 0 | 2 (6) | 0 | 2 (5) | 0 | **0.05** |
|  |  |  |  |  |  |  |  |
| **Stroke or TIA** | 5 (2) | 0 | 2 (6) | 0 | 3 (8) | 0 | **0.01** |
| **Periproc. resuscitation** | 8 (3) | 3 (3) | 3 (8) | 0 | 2 (5) | 0 | 0.21 |
| **Death before discharge** | 6 (2) | 1 (1) | 2 (6) | 2 (4) | 0 | 1 (6) | 0.31 |
| **30-d-mortality** |  |  |  |  |  |  |  |
|  |  |  |  |  |  |  |  |
| **Bleeding complications*** |  |  |  |  |  |  |  |
| - **Leading to death (type 4)** | 1 (0.4) | 0 | 1 (3) | 0 | 0 | 0 | 0.20 |
| - **Life-threatening (type 3)** | 6 (2) | 1 (1) | 1 (3) | 0 | 1 (3) | 0 | 0.68 |
| - **Major (type 2)** | 72 (29) | 34 (32) | 6 (17) | 17 (33) | 11 (29) | 4 (24) | 0.45 |
| - **Minor bleeding (type 1)** | 47 (19) | 13 (12) | 11 (31) | 10 (19) | 8 (21) | 5 (29) | 0.08 |
| **Cardiac tamponade** | 2 (1) | 1 (1) | 1 (3) | 0 | 0 | 0 | 0.61 |
| **Hb before TAVI,** *g/dL* | 12.2±1.7 | 12.4±1.6 | 12.3±1.7 | 12.1±1.7 | 11.7±1.7 | 12.3±1.8 | 0.23 |
| **Drop in Hb post TAVI,** *g/dL* | 2.4±1.3 | 2.5±1.3 | 2.1±0.9 | 2.3±1.4 | 2.6±1.4 | 2.1±1.3 | 0.31 |
|  |  |  |  |  |  |  |  |
| **Acute kidney injury***  **(modified RIFLE classification*)** |  |  |  |  |  |  |  |
| - **Stage 1** | 28 (11) | 13 (12) | 3 (8) | 5 (10) | 7 (18) | 0 | 0.33 |
| - **Stage 2** | 3 (1) | 1 (1) | 0 | 1 (2) | 1 (3) | 0 | 0.81 |
| - **Stage 3** | 9 (4) | 3 (3) | 2 (6) | 2 (4) | 1 (3) | 1 (6) | 0.92 |
| **Patients requiring RRT** | 3 (1) | 1 (1) | 1 (3) | 1 (2) | 0 | 0 | 0.78 |
| **Creatinine before TAVI,** *mg/dL* | 1.3±1.3 | 1.1±0.6 | 1.3±1.0 | 1.9±2.4 | 1.4±0.8 | 1.2±0.5 | **<0.0001** |
| **Max. creatinine up to 72h after TAVI,** *mg/dL* | 1.5±1.2 | 1.3±1.0 | 1.4±1.3 | 1.9±1.8 | 1.6±1.1 | 1.3±0.7 | **0.001** |
|  |  |  |  |  |  |  |  |
| **Access-related complications*** |  |  |  |  |  |  |  |
| **Major access complications*** | 11 (4) | 3 (3) | 1 (3) | 3 (8) | 4 (11) | 0 | 0.26 |
| - **Thoracic aortic dissection** | 0 | 0 | 0 | 0 | 0 | 0 | NA |
| **Minor access complications*** | 64 (26) | 23 (21) | 12 (33) | 13 (25) | 11 (29) | 5 (29) | 0.66 |
| **Unplanned intervention** | 15 (6) | 5 (5) | 5 (14) | 4 (8) | 1 (3) | 0 | 0.17 |
| **Unplanned surgery** | 3 (1) | 2 (2) | 0 | 1 (2) | 0 | 0 | 0.78 |
|  |  |  |  |  |  |  |  |
| **New-onset conduction disturbances** |  |  |  |  |  |  |  |
| - **New LBBB** | 37 (15) | 21 (20) | 2 (6) | 5 (10) | 6 (16) | 3 (18) | 0.23 |
| - **Third degree AV block** | 25 (10) | 6 (6) | 5 (14) | 5 (10) | 3 (8) | 3 (18) | 0.37 |
| - **New permanent pacemaker** | 26 (10) | 11 (10) | 4 (11) | 7 (13) | 3 (8) | 1 (6) | 0.86 |
| **Supraventricular arrhythmias** | 16 (6) | 10 (9) | 1 (3) | 3 (6) | 2 (5) | 0 | 0.46 |
|  |  |  |  |  |  |  |  |
| **Valve endocarditis** | 0 | 0 | 0 | 0 | 0 | 0 | NA |
|  |  |  |  |  |  |  |  |

- * Definitions according to proposed endpoint definitions from the Valve Academic Research Consortium (VARC-3).
- Comparison of all 5 groups: Kruskal-Wallis-test for continuous variables in the absence of normality distribution; chi-square test for categorical variables

**Suppl. Table 3 A-D: Reverse remodelling in AS subtypes at 6 months after TAVI (only patients with matched observations)**

| **NEF-HG AS**  **FU 79% complete (85/107)** | | **Baseline**  **(n=85)** | **Follow-up (n=85)** | **P** |
| --- | --- | --- | --- | --- |
|  |  | |  |  |
| **Afib during echo,** *n (%)* | 8 (9%) | | 8 (9%) | 1 |
| **LV-EF,** *%* | 60±6 | | 59±6 | 0.33 |
| **Global long. strain,** *%* | -18.1±2.7 | | -18.5±2.8 | 0.37 |
| **LVEDV,** *ml* | 71±23 | | 68±19 | 0.15 |
| **LVEDV_i_,** *ml/m² BSA* | 37±10 | | 36±10 | 0.28 |
| **LVESV,** *ml* | 29±12 | | 28±10 | 0.36 |
| **Stroke Volume Index,** *ml/m²* | 41.5±8 | | 47.8±22 | **0.045*** |
| **LAVI,** *ml/m² BSA* | 44±12 | | 41±13 | **0.047*** |
| **LVMI,** *g/m² BSA* | 133±30 | | 115±23 | **<0.0001*** |
| **LVMI/ LVEDV_i_,** *g/ml* | 3.7±1.0 | | 3.3±1.0 | **0.003*** |
| **LVEDD,** *mm* | 41.8±6 | | 40.9±5 | 0.16 |
| **LVESD,** *mm* | 29.7±6 | | 26.5±6 | **0.0001*** |
| **Septal wall,** *mm* | 16.1±2.3 | | 14.9±1.9 | **<0.0001*** |
| **Posterior wall,** *mm* | 14.0±2.2 | | 12.9±1.7 | **<0.0001*** |
| **Relative wall thickness** | 0.69±0.15 | | 0.64±0.11 | **0.008*** |
| **Concentric remodelling,** *n (%)* | 11 (13%) | | 29 (35%) | **0.001*** |
| **Concentric hypertrophy,** *n (%)* | 72 (85%) | | 55 (65%) | **0.003*** |
| **Eccentric hypertrophy,** *n (%)* | 2 (2%) | | 0 | 0.15 |
| **E/e’ mean** | 18.0±7.0 | | 16.4±6.9 | 0.37 |
| **V_max_,** *m/s* | 4.5±0.5 | | 2.1±0.6 | **<0.0001*** |
| **Mean Gradient,** *mmHg* | 49±13 | | 10±7 | **<0.0001*** |
| **Aortic valve area (AVA),** *cm²* | 0.70±0.15 | | 1.77±0.54 | **<0.0001*** |
| **Indexed AVA,** *cm²/m² BSA* | 0.37±0.08 | | 0.94±0.28 | **<0.0001*** |
| **PAsP,** *mmHg* | 40.9±13 | | 35±9 | 0.07 |
| **TAPSE,** *mm* | 22.5±4.3 | | 22.3±4.9 | 0.53 |
| **Moderate or severe MR,** *n (%)* | 29 (35%) | | 12 (14%) | **0.002*** |
| **Moderate or severe TR,** *n (%)* | 14 (16%) | | 12 (14%) | 0.67 |
|  |  | |  |  |

- 2-group-comparisons: t-test for continuous variables; fisher’s exact test for categorical variables
- Afib: atrial fibrillation: LV-EF: left ventricular ejection fraction; LVEDV: left ventricular end-diastolic volume; BSA: body surface area; SVI: stroke volume index; LAVI: left atrial volume index; LVMI: left ventricular mass index; LVEDD: left ventricular end-diastolic diameter; RWT: relative wall thickness; GLS: global longitudinal strain; v_max_: maximum aortic velocity; AVA: aortic valve are; AVAi: indexed aortic valve area, AVA/BSA; PAsP: pulmonary artery systolic pressure; TAPSE: tricuspid annular plane systolic elevation; MR: mitral regurgitation; TR: tricuspid regurgitation

| **LEF-HG AS**  **FU 75% complete (27/36)** | | **Baseline**  **(n=27)** | **Follow-up (n=27)** | **P** |
| --- | --- | --- | --- | --- |
|  |  | |  |  |
| **Afib during echo,** *n (%)* | 5 (19) | | 5 (19) | 1.0 |
| **LV-EF,** *%* | 37±10 | | 52±13 | **<0.0001*** |
| **Global long. strain,** *%* | -11.1±3.7 | | -14.4±2 | **<0.0001*** |
| **LVEDV,** *ml* | 115±34 | | 85±29 | **<0.0001*** |
| **LVEDV_i_,** *ml/m² BSA* | 58±14 | | 44±16 | **<0.0001*** |
| **LVESV,** *ml* | 74±29 | | 42±16 | **<0.0001*** |
| **Stroke Volume Index,** *ml/m²* | 34.9±9 | | 35.7±9 | 0.58 |
| **LAVI,** *ml/m² BSA* | 61±20 | | 51±22 | **0.01*** |
| **LVMI,** *g/m² BSA* | 177±37 | | 142±38 | **<0.0001*** |
| **LVMI/ LVEDV_i_,** *g/ml* | 3.15±0.8 | | 3.42±0.9 | **<0.0001*** |
| **LVEDD,** *mm* | 51.9±9 | | 46.7±7 | **<0.0001*** |
| **LVESD,** *mm* | 42.6±9 | | 36.2±8 | **0.0002*** |
| **Septal wall,** *mm* | 15.9±2 | | 15.5±2 | 0.23 |
| **Posterior wall,** *mm* | 14.1±3 | | 13.2±2 | **0.02*** |
| **Relative wall thickness** | 0.57±0.18 | | 0.58±0.12 | 0.76 |
| **Concentric remodelling,** *n (%)* | 0 | | 5 (19) | **0.02*** |
| **Concentric hypertrophy,** *n (%)* | 20 (74) | | 19 (70) | 0.76 |
| **Eccentric hypertrophy,** *n (%)* | 7 (26) | | 3 (11) | 0.16 |
| **E/e’ mean** | 19.6±7 | | 16.7±7 | 0.08 |
| **V_max_,** *m/s* | 4.4±0.5 | | 2.2±0.3 | **<0.0001*** |
| **Mean Gradient,** *mmHg* | 48±11 | | 10±3 | **<0.0001*** |
| **Aortic valve area (AVA),** *cm²* | 0.62±0.16 | | 1.43±0.31 | **<0.0001*** |
| **Indexed AVA,** *cm²/m² BSA* | 0.32±0.08 | | 0.74±0.19 | **<0.0001*** |
| **PAsP,** *mmHg* | 54±18 | | 39±14 | **0.002*** |
| **TAPSE,** *mm* | 20±4 | | 22±4 | 0.18 |
| **Moderate or severe MR,** *n (%)* | 13 (48) | | 7 (26) | 0.09 |
| **Moderate or severe TR,** *n (%)* | 8 (30) | | 7 (26) | 0.76 |
|  |  | |  |  |

| **LEF-LG AS**  **FU 62% complete (32/52)** | | **Baseline**  **(n=52)** | **Follow-up (n=52)** | **P** |
| --- | --- | --- | --- | --- |
|  |  | |  |  |
| **Afib during echo,** *n (%)* | 6 (19) | | 6 (19) | 1.0 |
| **LV-EF,** *%* | 35±9 | | 45±12 | **<0.0001*** |
| **Global long. strain,** *%* | -10±4.6 | | -13.4±3.0 | **<0.0001*** |
| **LVEDV,** *ml* | 132±51 | | 112±51 | **0.02*** |
| **LVEDV_i_,** *ml/m² BSA* | 65±22 | | 56±25 |  |
| **LVESV,** *ml* | 89±44 | | 64±39 | **0.0003*** |
| **Stroke Volume Index,** *ml/m²* | 31.3±7 | | 30.8±8.6 | 0.61 |
| **LAVI,** *ml/m² BSA* | 50±12 | | 41±11 | **0.002*** |
| **LVMI,** *g/m² BSA* | 168±39 | | 136±29 | **<0.0001*** |
| **LVMI/ LVEDV_i_,** *g/ml* | 2.73±0.8 | | 2.77±1.1 | **0.01*** |
| **LVEDD,** *mm* | 54.8±8 | | 50.7±7.8 | **0.0001*** |
| **LVESD,** *mm* | 45.0±8 | | 40.6±9.3 | **0.004*** |
| **Septal wall,** *mm* | 15.0±3 | | 13.8±2.1 | **0.01*** |
| **Posterior wall,** *mm* | 12.9±2 | | 12.4±1.8 | 0.24 |
| **Relative wall thickness** | 0.49±0.13 | | 0.50±0.12 | 0.38 |
| **Concentric remodelling,** *n (%)* | 0 | | 5 (16) | **0.02*** |
| **Concentric hypertrophy,** *n (%)* | 17 (53) | | 18 (58) | 0.77 |
| **Eccentric hypertrophy,** *n (%)* | 15 (47) | | 6 (19) | **0.01*** |
| **E/e’ mean** | 13.8±4 | | 14.7±5.2 | 0.47 |
| **V_max_,** *m/s* | 3.3±0.4 | | 2.0±0.5 | **<0.0001*** |
| **Mean Gradient,** *mmHg* | 26±7 | | 8.8±3.9 | **<0.0001*** |
| **Aortic valve area (AVA),** *cm²* | 0.80±0.15 | | 1.67±0.26 | **0.0002*** |
| **Indexed AVA,** *cm²/m² BSA* | 0.40±0.08 | | 0.79±0.09 | **<0.0001*** |
| **PAsP,** *mmHg* | 49±13 | | 37±11 | **0.003*** |
| **TAPSE,** *mm* | 18±4 | | 18±5 | 0.63 |
| **Moderate or severe MR,** *n (%)* | 21 (66) | | 11 (41) | **0.006*** |
| **Moderate or severe TR,** *n (%)* | 10 (31) | | 6 (22) | 0.23 |
|  |  | |  |  |

| **PLF-LG AS**  **FU 63% complete (24/38)** | | **Baseline**  **(n=24)** | **Follow-up (n=24)** | **P** |
| --- | --- | --- | --- | --- |
|  |  | |  |  |
| **Afib during echo,** *n (%)* | 13 (54) | | 10 (42) | 0.39 |
| **LV-EF,** *%* | 58±6 | | 58±7 | 0.88 |
| **Global long. strain,** *%* | -16.8±2.5 | | -17.78±2.0 | 0.05 |
| **LVEDV,** *ml* | 62±26 | | 54±1 | 0.06 |
| **LVEDV_i_,** *ml/m² BSA* | 34±11 | | 30±7 | 0.11 |
| **LVESV,** *ml* | 27±13 | | 23±7 | 0.05 |
| **Stroke Volume Index,** *ml/m²* | 30.5±4.0 | | 35.1±11.6 | 0.09 |
| **LAVI,** *ml/m² BSA* | 54±16 | | 53±17 | 0.85 |
| **LVMI,** *g/m² BSA* | 131±29 | | 124±26 | 0.16 |
| **LVMI/ LVEDV_i_,** *g/ml* | 4.2±1.3 | | 4.36±1.5 | 0.56 |
| **LVEDD,** *mm* | 43.1±5.2 | | 42.2±5.2 | 0.37 |
| **LVESD,** *mm* | 32.0±6.9 | | 29.2±6.1 | **0.045*** |
| **Septal wall,** *mm* | 14.6±2.1 | | 14.2±2.0 | 0.23 |
| **Posterior wall,** *mm* | 13.3±1.9 | | 13.2±2.1 | 0.80 |
| **Relative wall thickness** | 0.62±0.12 | | 0.64±0.14 | 0.69 |
| **Concentric remodelling,** *n (%)* | 2 (8) | | 6 (25) | 0.12 |
| **Concentric hypertrophy,** *n (%)* | 22 (92) | | 18 (75) | 0.12 |
| **Eccentric hypertrophy,** *n (%)* | 0 | | 0 | 1.0 |
| **E/e’ mean** | 15.1±5.5 | | 15.96±6.6 | 0.98 |
| **V_max_,** *m/s* | 3.2±0.3 | | 2.0±0.4 | **<0.0001*** |
| **Mean Gradient,** *mmHg* | 24.1±5.2 | | 9.5±4.0 | **<0.0001*** |
| **Aortic valve area (AVA),** *cm²* | 0.76±0.14 | | 1.58±0.39 | **<0.0001*** |
| **Indexed AVA,** *cm²/m² BSA* | 0.43±0.10 | | 0.87±0.21 | **<0.0001*** |
| **PAsP,** *mmHg* | 51±21 | | 39±13 | **0.03*** |
| **TAPSE,** *mm* | 19±4 | | 19±5 | 0.9 |
| **Moderate or severe MR,** *n (%)* | 13 (54) | | 9 (37) | 0.25 |
| **Moderate or severe TR,** *n (%)* | 11 (46) | | 10 (42) | 0.77 |
|  |  | |  |  |
